# Evaluation of Contrast Sensitivity, Chromatic Vision, and Reading Ability in Patients with Primary Open Angle Glaucoma

**DOI:** 10.1155/2016/7074016

**Published:** 2016-10-31

**Authors:** Maria P. Bambo, Blanca Ferrandez, Noemi Güerri, Isabel Fuertes, Beatriz Cameo, Vicente Polo, Jose M. Larrosa, Elena Garcia-Martin

**Affiliations:** ^1^Miguel Servet University Hospital, Ophthalmology Department, Zaragoza, Spain; ^2^Aragon Institute for Health Research (IIS Aragón), University of Zaragoza, Zaragoza, Spain

## Abstract

*Purpose*. To compare contrast sensitivity, acquired color vision deficiency, and reading ability in patients with glaucoma at different stages of the disease and to establish correlations between visual field parameters and visual function scores.* Methods*. This prospective cross-sectional study included 121 glaucoma patients. Subjects with a diagnosis of chronic open angle glaucoma were recruited and classified according to Hodapp-Parrish-Anderson criteria. Patients with severe visual field defects were excluded because they were older, which could bias the interpretation of visual function tests. Contrast sensitivity was measured using the Pelli-Robson Chart and the CSV1000E test. Chromatic vision was evaluated using the Farnsworth-panel D15 and the L'Anthony D15 tests of Vision Color Recorder software. Reading ability was measured using Radner-Vissum test.* Results*. Contrast sensitivity (with photopic and mesopic luminance with glare) differed significantly between patients with early and moderate visual field defects (*p* < 0.05). Reading ability scores and results of the chromatic vision tests did not differ significantly between the two groups. Significant and moderate Spearman correlations between visual field indexes and contrast sensitivity tests were detected.* Conclusions*. Contrast sensitivity was significantly worse in patients with moderate glaucoma compared to those with early-stage glaucoma. Evaluation of visual function in clinical practice provides important information to address a glaucoma patient's vision complaints.

## 1. Introduction

Vision loss due to glaucoma is traditionally described as a loss of “peripheral vision” [1], although this is not the most commonly reported symptom. Requiring more light and blurry vision are the most common complaints of patients with glaucoma [2].

Even from early stages of the disease, glaucoma patients often complain of much worse vision than we would expect based on their good visual acuity; and this apparent discrepancy may be based on the decrease in visual function caused by glaucoma. Visual ability in low illumination conditions and the capability to detect low-contrast objects are two important functions in daily life of patients with peripheral vision loss due to glaucoma [3]. Recent studies suggest that glaucoma is also associated with greater self-reported reading difficulty [4] and modestly lower reading speeds [5]. Color vision defects in patients with glaucoma, especially along the blue-yellow axis, are also reported [6].

The goal of this study was to evaluate a large sample of glaucoma patients at different stages of disease severity to further investigate contrast sensitivity, color vision, and reading ability using various psychophysical tests. To the best of our knowledge, there are no studies that evaluate contrast sensitivity in glaucoma patients under different lighting conditions (photopic and mesopic with glare condition, which could resemble night driving). In addition, we evaluated the correlation between visual function worsening and visual field damage in glaucoma. Characterizing visual function in patients with glaucoma (beyond the visual field) may be useful toward enhancing our knowledge and developing potential methods for enhancing vision-related function in these patients.

## 2. Materials and Methods

The local ethics committee approved the study procedure, which was conducted in accordance with the Declaration of Helsinki. Informed consent was obtained from all participants.

### 2.1. Study Subjects

All glaucoma patients were recruited from the Glaucoma Department at the Miguel Servet University Hospital (Zaragoza, Spain).

Inclusion criteria included a clinical diagnosis of glaucoma at a previous visit at least 1 year before. Patients with primary open angle glaucoma, normal-tension glaucoma, pseudoexfoliative glaucoma, or pigment dispersion glaucoma were included. A diagnosis of glaucoma was based on characteristic optic nerve damage on slit-lamp examination (defined as a definite notch in the neuroretinal rim or absence of the neuroretinal rim not due to another known cause) with corresponding visual field defects. Only eyes with best corrected visual acuity (BCVA) of 20/30 or better based on the Snellen chart were included.

Subjects were excluded if they had vision loss secondary to another eye condition, had any laser procedure in the previous month, or had any ocular surgery in the previous 3 months. Other exclusion criteria included extreme refractive errors such as high myopia (−5.0 or higher), hyperopia (+5.0 or higher), or astigmatism (±3.0 or higher) or acute angle closure glaucoma, congenital color vision defects, or history of stroke or neurologic pathology. Patients with clinically significant lenticular opacity using the LOCS III classification [7] were also excluded. The exclusion criteria for lenticular opacity were nuclear color/opalescence greater than NC2 and NO2, respectively, cortical cataract greater than C2, and posterior subcapsular cataract greater than or equal to P1.

### 2.2. Ophthalmological Examination

The ocular examination included measurements of BCVA using ETDRS Charts (Precision Vision, IL, USA) at 4 m with decimal Snellen visual acuity notation and intraocular pressure (IOP) (using a calibrated Goldmann applanation tonometer), slit-lamp examination of the anterior segment, and fundus evaluation. Given the influence of pupil size in psychophysical testing [8], horizontal pupil diameter measurements were obtained using a Colvard pupillometer (Oasis Medical, Glendora, California) in photopic lighting at 120 candelas/m^2^ (cd/m^2^). The Humphrey 24-2 Swedish Interactive Threshold Algorithm Standard perimeter (Zeiss Meditec, Dublin, CA) was used to evaluate the visual field. All enrolled subjects were classified according to two stages of visual field damage using the Hodapp-Parrish-Anderson criteria [9]: early and moderate defects (Table 1). Patients with severe defects were excluded because they were older, which could bias the interpretation of visual function tests.

### 2.3. Contrast Sensitivity Evaluation

The Pelli-Robson chart test and the CSV-1000E were used to evaluate contrast sensitivity. The Pelli-Robson test is a contrast sensitivity test with 8 lines and 16 levels of horizontal capital letters measuring central vision. Each set of three letters on the chart becomes progressively lower in contrast relative to the background. Each eye is evaluated separately with the nontested eye occluded. The chart is mounted on a white wall with the patient sitting 1 meter away from the chart; the luminance of the test is photopic, at 120 cd/m^2^, with the accepted range being between 60 and 120 cd/m^2^ [10].

The CSV-1000E chart (Vector Vision, Haag-Streit, Harlow, UK) is a contrast sensitivity chart test with four rows of sine-wave gratings. At the recommended test distance of 2.5 m, these gratings test the spatial frequencies of 3, 6, 12, and 18 cycles/degree (cpd) to establish a functional acuity score for patients under nonglare and glare conditions [11]. Each eye is evaluated separately with the nontested eye occluded. A functional acuity score can be obtained directly from the test-scoring sheet, and log values were selected. Ambient lighting used to conduct the test was photopic (120 cd/m^2^) without glare and mesopic (0.8 cd/m^2^) with glare condition (after 10 min of dark adaptation, at 0.8 cd/m^2^).

### 2.4. Color Vision Evaluation

The Farnsworth-panel D15 (F-D15) and the L'Anthony desaturated D15 (L-D15) tests of Vision Color Recorder software (Optical Diagnosis, Inc., Beusichem, Netherlands) were performed in all the study subjects using the best corrected refraction for near sight under specific photopic lighting (120 cd/m^2^) and at a 45° angle to the screen. Each eye was evaluated separately with the nontested eye occluded. F-D15 and L-D15 are both arrangement tests in which the subject is offered a series of colors that need to be sorted either into a sequence (usually based on hue) or into groups (most often grays versus colors). The L-D15 test is more sensitive than the F-D15 test for detecting color vision impairment. Therefore, a subject who passes the F-D15 test but fails the L-D15, which has smaller color differences, may have a mild defect in chromatic vision.

For grading acquired color vision defects, we used the Confusion Color Index (CCI) developed by Bowman [12]. CCI assesses the severity of dyschromatopsia. A CCI score higher than 1 indicates altered color vision perception; the higher the score, the worse the condition.

### 2.5. Reading Ability Evaluation

Radner et al. developed the Radner reading charts, which are based on the concept of using sentence optotypes for the standardized examination of reading acuity and reading speed [13]. The Spanish version of the Radner reading charts, Radner-Vissum test, was administered to all of the study patients binocularly [14]. The reading distance was 40 cm. A standard luminance of 80 cd/m^2^ was used.

The parameters registered were LogRAD score and maximum reading speed (MRS) in words per minute (wpm). LogRAD score is a correction of the reading acuity (LogRAD) for the number of reading errors (LogRAD score = LogRAD + 0.005 × syllables of incorrectly read words). The reading speed was calculated based on the number of words in a sentence and the time needed to read the sentence (14 words × 60 seconds divided by the reading time = words per minute). The mean reading speed of the subsequent sentences up to the Critical Print Size was defined as the MRS. The Critical Print Size is the print size at which reading speed begins to deteriorate; after calculating the reading speed per sentence (wpm), this was plotted on a graph and the print size where the line suddenly dropped was defined as the Critical Print Size.

### 2.6. Statistical Analysis

This was an observational, prospective cross-sectional study. A total of 121 eyes of 121 patients were included in the statistical analysis. One eye from each subject was randomly chosen for the study, unless only one eye met the inclusion criteria. All 121 eyes were stratified according to the severity of glaucomatous damage in two stages (early and moderate defects) following Hodapp-Parrish-Anderson criteria [9] (Table 1). All data analyses were performed using SPSS version 20.0 (SPSS Incorporation, Chicago, IL) statistical software. The Kolmogorov-Smirnov test was used to assess sample distribution. Due to the nonparametric distribution of the data, the results were compared using the Mann-Whitney nonparametric test. Correlations were examined by Spearman's test. A *p* value of 0.05 was considered statistically significant.

## 3. Results

One hundred and twenty-one eyes of 121 glaucoma patients were examined (94 eyes with early defects and 27 with moderate defects). Epidemiologic and disease characteristics of each of the two groups of patients are shown in Table 2. Sixty-four patients were females (52.9%) and 57 were males (47.1%). The two groups did not differ with respect to age or sex. Pupil size did not differ between the groups.

The visual function results are summarized in Table 3. The contrast sensitivity evaluation indicated statistical differences of the Pelli-Robson and CSV1000E parameters (photopic luminance; all frequencies) between patients with early and moderate defects (*p* < 0.05). The CSV1000E test performed with mesopic luminance and glare conditions also revealed statistically significant differences in all frequencies except 12 cpd between patients with early and moderate defects (*p* < 0.05). Our analysis revealed no significant differences in color vision and reading ability between the early and moderate defect groups.

Correlations between visual field indexes and contrast sensitivity tests were moderate and statistically significant (Table 4). The highest correlations were between the Pelli-Robson results and mean deviation (*r* = 0.43, *p* < 0.001); Pelli-Robson and pattern standard deviation (*r* = −0.48, *p* < 0.001); and Pelli-Robson and visual field index (*r* = 0.38, *p* < 0.001). Correlations were lower (*r* absolute value ranged between 0.25 and 0.39), but statistically significant, between almost all variables of the visual field indexes (especially mean deviation) and CSV1000E results performed in photopic luminance and mesopic luminance with glare, with the best correlations in the low frequencies (3 and 6 cpd). Visual field indexes and reading ability results were not significantly correlated. The visual field indexes and CCI were not significantly correlated, except for pattern standard deviation and CCI of L-D15 with a significant weak correlation (*r* = 0.21, *p* = 0.019).

## 4. Discussion

Although visual field measurement and optic disc evaluation are the principal methods for confirming the presence and progression of glaucoma, psychophysical tests to examine the function of specific parts of the visual pathway are also useful for monitoring glaucomatous changes. The decline in spatial contrast sensitivity in patients with glaucoma has been documented in different tests [15]. The Pelli-Robson chart produces reliable and reproducible results [16]. The Pelli-Robson chart tests the spatial frequency of 1 cpd at a distance of 1 meter. Patients with ocular hypertension and glaucoma demonstrate contrast sensitivity losses at spatial frequencies between 0.25 and 8 cpd [17]. Our findings indicated that contrast sensitivity was worse in glaucoma patients with moderate defects than in patients with early defects, especially at low frequencies (<6 cpd), for both photopic and mesopic luminance with glare lighting. There was a moderate correlation between worsening visual field indexes and decreased contrast sensitivity, stronger with the Pelli-Robson than the CSV1000E test. Our result is consistent with other findings, such as those by Hawkins et al. [18] and Wilensky et al. [19], who reported a significant correlation between the mean deviation as measured with the Humphrey perimeter and the Pelli-Robson contrast sensitivity scores (*r* = 0.56, *p* < 0.001, *n* = 127; *r* = 0.59, *p* < 0.001, *n* = 120, resp.). Others report that contrast sensitivity can be more affected than high-contrast visual acuity in glaucoma patients [20, 21]. Although previous studies did not provide enough evidence to support the use of contrast sensitivity for the early detection of glaucoma [22, 23], contrast sensitivity correlates with the perimeter deviation [20, 21] and we believe that contrast sensitivity in conjunction with visual field testing will be a promising method of detecting functional changes in glaucoma patients—even in those with good visual acuity.

Regarding color vision examination, CCI obtained in the F-D15 and L-D15 tests did not differ among groups. Another study in patients with primary open angle glaucoma reported a prevalence of 60% for blue-yellow defects in contrast to only 3% for red-green defects [24]. Several theories have been introduced to explain tritan-like defects in glaucoma, such as a greater susceptibility of blue-yellow sensitive ganglion cells to IOP-related damage due to their morphology and connectivity to second-order neurons [25]. Our results suggest that patients with glaucoma manifest signs of deteriorating color discrimination ability (CCI > 1 in both groups), but we did not detect differences in color discrimination according to disease severity or a significant correlation between the results of color perception testing and visual field indexes. Moreover, age could have a potentially strong effect on the deterioration of CCI, as suggested in Vingrys and King-Smith [26].

Differences in reading ability were also not detected between groups based on severity in our study, and we detected no significant correlation with visual field indexes and reading scores. Several studies have demonstrated reading impairments in subjects with glaucoma through performance-based reading testing different from the Radner-Vissum test [5, 27]. Performance-based reading tests are commonly measured in strictly controlled conditions, which do not reflect the typical daily environment of the patient, and this kind of test could be subject to wide variability. Glaucoma patients often complain about problems with reading, especially small print, but the reading impairment reported in glaucoma patients [28] may be due to a number of mechanisms, including poor detection of low-contrast stimuli or inadequate lighting conditions, and this must be taken into account in clinical practice to suggest methods to enhance reading ability (creating proper lighting to optimize contrast or teaching strategies to mitigate fatigue). Considering the fact that in glaucoma visual acuity is preserved until advanced stages, it is expectable that reading ability is not affected so much for a long time. Further studies examining reading ability under dim light conditions and including patients with severe visual field defects are required to clarify this.

A possible limitation of our study is the presence of cataracts. We assume that the influence of cataracts in visual function measurements in this study was small because we excluded patients with BCVA worse than 20/30 or if they had clinically significant lenticular opacity based on the LOCS III classification [7]. Another limitation of the study was the difference in group size, which contributed to the data not conforming to the normal distribution and, consequently, a reduction in the statistical power that decreased the possibility of detecting differences.

The findings of the present study indicate that contrast sensitivity is more decreased as the severity of glaucoma increases, and this could account for the common complaint of patients with glaucoma of hazy or blurry vision despite good visual acuity. Contrast sensitivity was more closely related than color perception or reading ability with disease severity. The ability to distinguish contrast plays an important role in patients' everyday vision, and several tests are available to distinguish contrast [29]. The Pelli-Robson chart test is a low-technology, inexpensive method for measuring spatial contrast sensitivity, which can detect worsening visual function in glaucoma patients. We recommended its application to document worsening visual function in glaucoma patients. Changes in contrast sensitivity are a potential indicator of the level of glaucoma damage; however, prospective studies are required.

## Figures and Tables

**Table 1:** Hodapp-Parrish-Anderson classification system.

Early defect	Moderate defect	Severe defect
Mean deviation between 0 and −6 dB, AND at least one of the following listed criteria:	Mean deviation between −6 and −12 dB, AND at least one of the following listed criteria:	Mean deviation <−12 dB, AND at least one of the following listed criteria:

(i) A cluster of ≥3 points on the pattern deviation plot in an expected location of the visual field depressed below the 5% level, at least one of which is depressed below the 1% level, OR (ii) Pattern standard deviation significant at *p* < 0.05, OR (iii) Glaucoma Hemifield Test = Outside Normal Limits.	(i) ≥25% but <50% of points on the pattern deviation plot depressed below the 5% level, and ≥15% but <25% of points depressed below the 1% level, OR (ii) At least 1 point within the central 5° with sensitivity of <15 dB, but no points in the central 5° can have a sensitivity of 0 dB, OR (iii) Only one hemifield may have a point with sensitivity of <15 dB within 5° of fixation.	(i) ≥50% but <75% of points on pattern deviation plot depressed below the 5% level and ≥25% but <50% of points depressed below the 1% level, OR (ii) At least one point in the central 5° has a sensitivity of 0 dB, OR (iii) Points within the central 5° with sensitivity <15 dB in both hemifields.

dB: decibels.

**Table 2:** Descriptive and clinical data for 121 glaucoma patients classified into two groups according to disease severity (Hodapp-Parrish-Anderson criteria).

	Early defect (*n* = 94)	Moderate defect (*n* = 27)	^*∗*^ *p*
	Mean ± SD	Mean ± SD	
Age (years)	65.13 ± 9.37	66.44 ± 10.03	0.253
BCVA (decimal notation)	1.07 ± 0.23	0.94 ± 0.20	**0.016**
IOPb (mm Hg)	22.03 ± 2.86	22.37 ± 3.80	0.647
CDR	0.58 ± 0.19	0.75 ± 0.17	**<0.001**
MD (dB)	−1.95 ± 1.42	−8.74 ± 1.79	**<0.001**
PSD (dB)	2.19 ± 1.31	8.98 ± 2.49	**<0.001**
VFI (%)	97.23 ± 3.02	78.08 ± 6.22	**<0.001**
CP (*µ*m)	552.72 ± 41.80	545.92 ± 32.49	0.239
Photopic pupil size (mm)	3.29 ± 0.78	3.27 ± 0.63	0.803

^*∗*^ *p*: level of statistical significance in comparison between groups using the Mann-Whitney nonparametric test. Bold text shows the statistically significant results (*p* < 0.05).

BCVA: best corrected visual acuity; IOPb: basal intraocular pressure; CDR: cup-to-disc ratio; MD, PSD, and VFI, mean deviation (MD), pattern standard deviation (PSD), and visual field index (VFI) obtained in the visual field using Humphrey 24-2 Swedish Interactive Threshold Algorithm Standard perimeter; dB: decibels; CP: corneal pachymetry; *µ*m: micrometers; SD: standard deviation.

**Table 3:** Mean and standard deviation obtained in visual function tests with subjects classified into two groups according to disease severity (Hodapp-Parrish-Anderson criteria).

	Early defect (*n* = 94)	Moderate defect (*n* = 27)	*p* ^*∗*^
	Mean ± SD	Mean ± SD	
Pelli-Robson (F)	1.76 ± 0.18	1.51 ± 0.30	**<0.001**
CSV (F) 3 cpd	1.70 ± 0.22	1.51 ± 0.26	**0.003**
CSV (F) 6 cpd	1.87 ± 0.24	1.68 ± 0.29	**0.003**
CSV (F) 12 cpd	1.45 ± 0.30	1.24 ± 0.37	**0.022**
CSV (F) 18 cpd	1.02 ± 0.32	0.73 ± 0.35	**0.002**
CSV (M + G) 3 cpd	1.48 ± 0.31	1.25 ± 0.34	**0.001**
CSV (M + G) 6 cpd	1.29 ± 0.56	0.88 ± 0.61	**0.003**
CSV (M + G) 12 cpd	0.73 ± 0.56	0.46 ± 0.42	0.062
CSV (M + G) 18 cpd	0.40 ± 0.39	0.18 ± 0.21	**0.020**
LogRad score (RV)	0.09 ± 0.10	0.11 ± 0.15	0.668
MRS (wpm)	195.6 ± 36.1	189.4 ± 62.1	0.110
CCI F-D15	1.16 ± 0.24	1.12 ± 0.20	0.733
CCI L-D15	1.36 ± 0.33	1.43 ± 0.27	0.173

^*∗*^ *p*: level of statistical significance in comparison between groups using Mann-Whitney nonparametric test. Bold text shows the statistically significant results (*p* < 0.05).

RV: Radner-Vissum reading test; MRS: maximum reading speed; wpm: words per minute; F: photopic luminance; CSV: CSV1000E contrast sensitivity test; M + G: mesopic luminance with glare condition; cpd: cycles per degree (spatial frequency); CCI: confusion color index; F-D15: Farnsworth-panel D15; L-D15: L'Anthony desaturated D15; SD: standard deviation.

**Table 4:** Spearman correlation coefficients (*r*) between visual field parameters and contrast sensitivity, reading ability, and color vision measures.

	Mean deviation		Pattern standard deviation		Visual field index	
	*r*	*p* ^*∗*^	*r*	*p* ^*∗*^	*r*	*p* ^*∗*^
BCVA (Snellen)	0.38	**<0.001**	−0.47	**<0.001**	0.31	**<0.001**
Pelli-Robson (F)	0.43	**<0.001**	−0.48	**<0.001**	0.38	**<0.001**
CSV (F) 3 cpd	0.37	**<0.001**	−0.34	**0.008**	0.26	**0.016**
CSV (F) 6 cpd	0.38	**<0.001**	−0.27	**0.036**	0.34	**0.002**
CSV (F) 12 cpd	0.31	**0.003**	−0.21	0.115	0.33	**0.003**
CSV (F) 18 cpd	0.32	**0.002**	−0.12	0.358	0.32	**0.005**
CSV (M + G) 3 cpd	0.32	**0.001**	−0.39	**<0.001**	0.33	**<0.001**
CSV (M + G) 6 cpd	0.34	**<0.001**	−0.28	**0.032**	0.26	**0.023**
CSV (M + G) 12 cpd	0.25	**0.014**	−0.25	0.060	0.18	0.096
CSV (M + G) 18 cpd	0.30	**0.005**	−0.37	**0.005**	0.22	0.058
LogRad score (RV)	−0.05	0.787	0.33	0.184	−0.11	0.593
MRS (RV)	0.28	0.201	−0.39	0.067	0.27	0.654
CCI F-D15	−0.03	0.774	0.001	0.991	0.033	0.774
CCI L-D15	−0.16	0.144	0.21	**0.019**	−0.18	0.115

^*∗*^ *p*: level of statistical significance in Spearman correlations. Bold text shows the statistically significant results (*p* < 0.05).

BCVA: best corrected visual acuity; F: photopic luminance; CSV: CSV1000E contrast sensitivity test; M + G: mesopic luminance with glare condition; cpd: cycles per degree (spatial frequencies); RV: Radner-Vissum reading test; MRS: maximum reading speed; CCI: confusion color index; F-D15: Farnsworth-panel D15; L-D15: L'Anthony desaturated D15.
